# Observer-based optimal and robust output regulation for nonlinear systems

**DOI:** 10.1371/journal.pone.0355711

**Published:** 2026-08-19

**Authors:** Arifa K. A. Qazi, Attaullah Y. Memon

**Affiliations:** Department of Electronics and Power Engineering, PNEC, National University of Sciences & Technology (NUST), Islamabad, Pakistan; Air University, PAKISTAN

## Abstract

This paper investigates the output regulation problem using an observer-based inverse optimal controller within the nonlinear servomechanism framework for asymptotic convergence to desired references and rejection of time-varying disturbances generated by an exosystem. To address the practical constraint of full-state measurements, the system’s internal and external state estimation is done via a full-order high-gain observer. These estimated states are incorporated into the inverse optimal controller augmented with a conditional servocompensator within the Lyapunov redesign and saturated high-gain feedback framework to enhance transient performance and achieve asymptotic steady-state regulation. The proposed control scheme combines the optimality and robustness properties offered by the state feedback controller with disturbance rejection and state estimation in an output-feedback framework. The proposed output-feedback controller is validated on a nonlinear DC motor using MATLAB/Simulink. Finally, comparative simulations against baseline controllers further exhibit the robustness, performance, and practical feasibility of the proposed controller for high-performance control tasks.

## 1. Introduction

The nonlinear output regulation problem is a long-standing and fundamental challenge in the field of control theory, extensively explored owing to its wide-ranging applications in robotics, electric drives, aerospace, and process industries. The objective is to design a controller that ensures that the system outputs asymptotically track desired reference signals and effectively reject disturbances, all of which may be generated by an autonomous exosystem. These reference signals and disturbance are often time-varying and unmeasurable, posing significant challenges for feedback design and necessitating robust, optimal, and practical output regulation strategies. Key contributions addressing this challenge can be found in [1–3]. More recently, the output regulation problem has been addressed using several innovative strategies such as adaptive and learning based frameworks [4–6], sliding mode approaches [7], and model-based control techniques [8–10]. These advancements highlight the need for controller designs that integrate robustness, optimality, and practicality for nonlinear systems.

The nonlinear servomechanism framework offers a viable strategy for resolving the output regulation problem. In this framework, the controller incorporates a model of the external inputs to guarantee asymptotic tracking and disturbance rejection [11]. Initially proposed for constant exogenous inputs, the framework has since been extended to handle time-varying signals and disturbances [12–14]. In particular, Memon et al. [14] addressed this challenge using a conditional servocompensator augmented with a stabilizing controller designed within the Lyapunov-based saturated high-gain feedback structure to improve transient performance and eliminate steady-state regulation error. A key benefit of this technique is the freedom to select any suitable state feedback controller for stabilization allowing integration with advanced control strategies such as optimal control. Memon, in [15], further demonstrated the feasibility of combining inverse optimal control with conditional servocompensators, motivating further study into output regulation frameworks that embed additional optimality and robustness properties.

Techniques for optimal and inverse optimal control are well-established in literature and have been applied to problems such as stabilization, tracking, and output regulation across various domains [16–20]. In particular, nonlinear optimal control necessitates computing the solution to the Hamilton-Jacobi-Bellman (HJB) equations which are nonlinear partial differential equations. This process is computationally expensive and and the HJB equations typically lack closed-form solutions. To address this limitation, Freeman and Kotoković proposed the inverse optimal controller [21] which is an approximate analytical approach that relates the solution of the HJB equations to the a positive semi-definite function, hereafter referred to as a Control Lypunov Function (CLF), instead of relying on traditional cost functionals. This facilitates the design of nonlinear optimal controllers that exhibit desirable properties such as optimality, robustness, and disturbance rejection in the resulting control scheme. However, a key challenge in this approach is the identification of an appropriate CLF, which remains a non-trivial task for nonlinear systems.

CLF-based approaches yield controllers with inherent robustness properties enabling them to automatically counteract imperfections such as model uncertainties, unmodeled dynamics, and parametric variations typically encountered in real-world nonlinear systems. In many practical settings, it is useful to further characterize these robustness properties through stability margins which provide insights into how the system responds to input uncertainties that do not alter the system’s relative degree, such as those introduced by simplified actuator modeling. These uncertainties are typically characterized as (i) time-invariant static uncertainties that affect the steady-state behavior of the system but do not introduce additional dynamics, and (ii) time-varying dynamic uncertainties that introduce additional dynamics and may degrade transient performance or even lead to system destabilization. Two key quantifiable margins include the sector margin and disk margin, defined in [22], which help quantify the system’s tolerance to input perturbations. The sector margin $\left(\kappa_{1},\kappa_{2}\right)$ characterizes robustness in the presence of static uncertainties, while the disk margin 𝒟(κ) provides a combined measure that effectively characterizes robustness under dynamic uncertainties. Moreover, a nonlinear system exhibiting a disk margin 𝒟(κ) implies sector margin (κ,∞).

An inherent constraint of inverse optimal control is the prerequisite for comprehensive knowledge of the system dynamics to implement state feedback. However, technical and economic constraints greatly impact the availability of required states, thereby complicating the implementation of the state feedback control law. This lack of direct state measurement poses a significant challenge. To address these issues, observers are integrated with state feedback controllers for asymptotic state estimations from output measurements [23–27]. In addition to overcoming the constraints associated with the state measurement, observers also provide additional properties such as robustness to measurement noise and disturbance rejection.

High-Gain Observers belong to a class of observers that utilize the separation principle in the design of the control scheme, making them particularly useful in scenarios where full state feedback is required but not all states are directly measurable. In such cases, HGOs permit an initial design of the state feedback control law and then replacement of the true states with their respective estimations from the observer to obtain the output feedback control scheme. Furthermore, in addition to providing accurate and robust estimates of unmeasureable states, HGOs provide additional robustness against bounded external disturbances, unmodeled dynamics and nonlinearities, and modeling uncertainties of the order 𝒪(ε) where ε is the observer design parameter. Additionally, they offer fast convergence and computational efficiency, making them a convenient choice in diverse scenarios, particularly for minimum-phase or differentially flat systems [28]. Although HGOs are sensitive to measurement noise, they are preferred due to their computational efficiency, minimal tuning requirements, and strong theoretical guarantees when applied to minimum-phase systems.

Recent advancements in nonlinear controls have investigated data-driven and adaptive approaches such as multilayer neuroadaptive reinforcement learning [29] and state-filtered disturbance rejection [30]. While these approaches have demonstrated positive results when dealing with unstructured uncertainties and non-smooth noise without requiring full structural knowledge, they often necessitate expensive computations for real-time weight updates, full-state measurements, or complex heuristic tuning for optimal performance, often guaranteeing only uniform ultimate boundedness. However, many industrial and safety-critical systems require a deterministic, Lyapunov-based framework that guarantees instantaneous stability and low computation overhead. In such cases, an HGO-based inverse-optimal controller within the nonlinear output regulation framework guarantees asymptotic stability and robustness with definite margins. By implementing a closed-form control law, this controller ensures optimal performance and practical feasibility, particularly in real-time situations where computational economy and guaranteed stability are paramount. Furthermore, such an approach is also scalable to higher-order systems where data-driven approaches may experience a ‘complexity explosion’.

This research builds upon the foundational theoretical framework of introducing an inverse optimal controller as the stabilizing controller within the nonlinear servomechanism framework established in [15]. It extends the framework to provide a complete, constructive synthesis of inverse optimal control and a robust regulator, demonstrating that the resulting state-feedback control law embeds additional properties of optimality and robustness within the framework. The proposed design also ensures asymptotic regulation under the influence of time-varying disturbances and matched uncertainties, thereby ensuring strong performance guarantees. The study further extends the framework to a complete output-feedback structure via the integration of a full-order high-gain observer. This eliminates the practical limitation of requiring the full-state vector for feedback, accurately reconstructing the plant dynamics using only output measurements. The integration of these components within an optimal output-feedback control framework is demonstrated to have optimality and robustness capabilities beyond those of conventional configurations.

**Fig 1:**
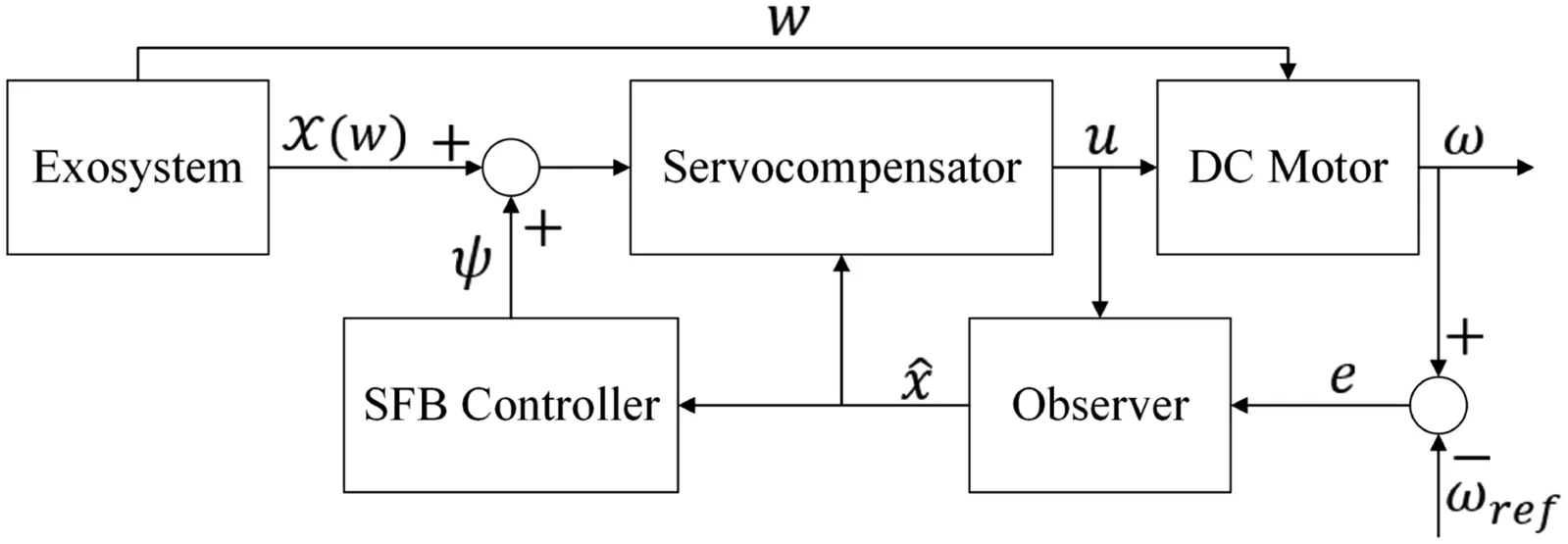
Proposed control scheme – Block diagram.

The primary outcome of this research is the integrated design, validation, and stability analysis of an HGO-based inverse optimal controller combined with a conditional servocompensator within the Lyapunov redesign framework, illustrated in Fig 1. In the figure, Ψ represents the stabilizing control signal, χ(w) is the steady-state control effort required to compensate for the disturbance *w*, and *u* represents the composite control signal into the plant. Furthermore, $\hat{x}$ represents the estimated state vector, ω is the mechanical speed of the motor, $\omega_{ref}$ is the desired reference speed, and *e* indicates the error between the actual speed and the desired reference.

The proposed control architecture is implemented on a nonlinear field-controlled DC motor, a benchmark exhibiting complex nonlinear dynamics, and validated through rigorous stress tests, including Monte Carlo simulations under parametric uncertainty. The results demonstrate improved transient performance, robust disturbance rejection, and optimal steady-state regulation, without necessitating the availability of the full-state vector, proving the viability of the proposed architecture for complex dynamical systems.

The remainder of the paper is structured as follows. Section 2 describes the system dynamics and formalizes the control problem under consideration. Section 3 focuses on the design of a state-feedback controller, later adapted to an output-feedback controller in Section 4. Section 5 presents the theoretical analysis followed by MATLAB/Simulink-based results in Section 6. Finally, the concluding remarks are presented in Section 7.

## 2. System modeling and problem formulation

The output regulation framework employing conditional servocompensators, as proposed by Khalil and coworkers [12,14], permits the integration of any stabilizing feedback controller for asymptotic tracking. We aim to extend this framework by leveraging this flexibility to embed additional properties of robustness and optimality into the overall control architecture.

As a representative case study, we consider a nonlinear field-controlled DC motor, a widely used system in industrial control applications. The system dynamics exhibit nonlinear coupling between the electrical and mechanical subsystems, making it a suitable test case for output feedback-based servocompensation design.

The mathematical model of the motor is expressed as [31]


$$\begin{array}{cc} \frac{di_{a}}{dt} & =-\frac{R_{a}}{L_{a}}i_{a}-\frac{c_{1}}{L_{a}}i_{f}\omega_{m}+\frac{v_{a}}{L_{a}}\\ \frac{di_{f}}{dt} & =-\frac{R_{f}}{L_{f}}i_{f}+\frac{1}{L_{f}}\;\nu (v_{f})\\ \frac{d\omega_{m}}{dt} & =\frac{c_{2}}{J}i_{a}i_{f}-\frac{c_{3}}{J}\omega_{m}\\ y & =\omega_{m} \end{array}$$ (1)

where $R_{a}$, $L_{a}$, $i_{a}$, and $v_{a}$ represent the armature resistance, inductance, current, and voltage, respectively; $R_{f}$, $L_{f}$, $i_{f}$, and $v_{f}$ represent the field resistance, inductance, current, and voltage, respectively, where $v_{f}$ is under the effect of a nonlinear perturbation ν(·); $\omega_{m}$ is the motor’s angular velocity, *J* is the rotor inertia, *c*_3_ is the damping coefficient, $c_{1}i_{f}\omega_{m}$ represents the back EMF, and $c_{2}i_{f}i_{a}$ represents the torque produced through the interplay of the armature current with the field circuit flux. Finally, *y* represents the output of the system.

**Assumption 1.** ν(μ) is a locally Lipschitz, sector-bounded nonlinearity satisfying


$$\begin{array}{c} \kappa_{1}\mu^{2}\;\leq \;\mu \;\nu (\mu )\;\leq \kappa_{2}\mu^{2},\;0<\kappa_{1}<\kappa_{2} \end{array}$$

**Remark 1.** Assumption 1 follows from the stability margins established for nonlinear systems in [22].

**Assumption 2.** A neutrally stable exosystem expressed as


$$\begin{array}{c} \dot{w}=S_{0}w \end{array}$$ (2)

generates the extrinsic signal $w(t)\in 𝒲\subset {\mathbb{R}}^{w}$, where *S*_0_ is characterized by unique eigenvalues located on the imaginary axis and is specified as


$$\begin{array}{c} S_{0}=[\begin{array}{ccc} 0 & 0 & 0\\ 0 & 0 & \omega \\ 0 & -\omega & 0 \end{array}],\;w=[\begin{array}{c} w_{1}\\ w_{2}\\ w_{3} \end{array}] \end{array}$$

$$\begin{array}{c} \left[\begin{array}{c} w_{1}\\ w_{2} \end{array}]=[\begin{array}{c} w_{ref}\\ d_{0}\sin(\omega t) \end{array}],\;w(0)=[\begin{array}{c} w_{ref}\\ d_{0} \end{array}\right] \end{array}$$

**Remark 2.** The requirement of the eigenvalues of S_0_ ensures the boundedness and persistence of w(t) in time. It also ensures that the system exhibits a steady-state response that is also persistent and depends on the specific input to the system instead of the initial states.

In the system (1), setting $\zeta_{1}=i_{a}$, $\zeta_{2}=i_{f}$, $\zeta_{3}=\omega_{m}$, $\mu =v_{f}$, $\alpha_{1}=\frac{R_{a}}{L_{a}}$, $\alpha_{2}=\frac{c_{1}}{L_{a}}$, $\alpha_{3}=\frac{v_{a}}{L_{a}}$, $\alpha_{4}=\frac{R_{f}}{L_{f}}$, $\alpha_{5}=\frac{1}{L_{f}}$, $\alpha_{6}=\frac{c_{2}}{J}$, and $\alpha_{7}=\frac{c_{3}}{J}$, and integrating it with the exosystem (2), we obtain


$$\begin{array}{cc} {\dot{\zeta }}_{1} & =-\alpha_{1}\zeta_{1}-\alpha_{2}\zeta_{2}\zeta_{3}+\alpha_{3}\\ {\dot{\zeta }}_{2} & =-\alpha_{4}\zeta_{2}+\alpha_{5}\left(\nu (\mu )+w_{2}\right)\\ {\dot{\zeta }}_{3} & =\alpha_{6}\zeta_{1}\zeta_{2}-\alpha_{7}\zeta_{3}\\ y & =\zeta_{3} \end{array}$$ (3)

**Assumption 3.** There exists a continuous mapping χ(w), originating from the internal model


$$\begin{array}{c} \frac{\partial \tau }{\partial w}S_{0}w=S\tau (w),\;\chi (w)=\Gamma \tau (w) \end{array}$$ (4)

where *S* is characterized by unique eigenvalues located on the imaginary axis. Furthermore, for a set of real numbers $\vartheta_{0},\vartheta_{1},\cdots ,\vartheta_{q-1}$, the function χ(w) satisfies


$$\begin{array}{c} {ℒ}_{s}^{q}\chi =\vartheta_{0}\chi +\vartheta_{1}{ℒ}_{s}\chi +\cdots +\vartheta_{q-1}{ℒ}_{s}^{q-1}\chi ,\;\forall \;w\in 𝒲 \end{array}$$ (5)

where ${ℒ}_{s}\chi =\frac{\partial \chi }{\partial w}S_{0}w$. Moreover, $q\in {\mathbb{Z}}^{+}$ indicates the dimension of the exosystem and is the degree of the minimal polynomial of *S* [14]:


$$\begin{array}{c} \lambda^{q}-\vartheta_{q-1}\lambda^{q-1}-\;\cdots \;-\vartheta_{0}=0 \end{array}$$

which exhibits unique roots restricted to the imaginary axis. This enables the definition of matrices


$$\begin{array}{c} S=[\begin{array}{ccccc} 0 & 1 & 0 & \cdots & 0\\ 0 & 0 & 1 & \cdots & 0\\ \vdots & \vdots & \vdots & \vdots & \vdots \\ 0 & 0 & \cdots & \cdots & 1\\ \vartheta_{0} & \vartheta_{1} & \cdots & \cdots & \vartheta_{q-1} \end{array}],\;\tau =[\begin{array}{c} \chi \\ L_{s}\chi \\ \vdots \\ L_{s}^{q-2}\chi \\ L_{s}^{q-1}\chi \end{array}],\;\Gamma^{T}={\left[\begin{array}{c} 1\\ 0\\ \vdots \\ 0 \end{array}\right]}_{1\times q} \end{array}$$ (6)

such that they conform to the criteria presented in [14].

**Remark 3.** The finite-dimensional linear internal model in (4) is derived from the foundational work on the nonlinear internal model principle [32–34] combined with the concept of immersion mapping [35], and is valid only when χ(w) contains a finite number of harmonics, such as when χ(w) is a polynomial function of w. This additional condition ensures that χ(w) is finite-dimensional, allowing the infinite-dimensional output regulation problem to be reduced to a practically implementable finite-dimensional linear servocompensator design defined by (4)-(6).

**Assumption 4.** For the system (3), which can be characterized as


$$\begin{array}{cc} \dot{\zeta } & =\phi_{\zeta }(\zeta ,w)+\gamma_{\zeta }(\zeta ,w)\mu \\ e & =h_{\zeta }(\zeta ,w) \end{array}$$

a continuously differentiable map $\zeta_{w}=\Pi (w)$ exists for all w∈𝒲, fulfilling Π(0)=0, and a continuous function χ(w) which solves ∀ w∈𝒲,


$$\begin{array}{cc} \frac{\partial \Pi (w)}{\partial w}S_{0}w & =\phi_{\zeta }(\Pi ,w)+\gamma_{\zeta }(\Pi ,w)\chi (w)\\ e & =h_{\zeta }(\Pi ,w)=0 \end{array}$$ (7)

**Remark 4.** Assumption 4 establishes $\zeta_{w}=\Pi (w)$ as the zero-error invariant manifold and on which the trajectories are maintained, in the presence of exogenous input w, through the steady-state control χ(w). This is a necessary and sufficient condition for solving the output regulation problem.

Therefore, using the state variable transformation $[z,x{]}^{T}=\zeta -\Pi$, the system (3) can be reformulated as


$$\begin{array}{c} \dot{z}=-\alpha_{1}z-\alpha_{2}x_{1}(x_{2}+w_{1})+\alpha_{3} \end{array}$$ (8a)

$$\begin{array}{c} {\dot{x}}_{1}=-\alpha_{4}x_{1}+\alpha_{5}(\nu (\mu )+w_{2}) \end{array}$$ (8b)

$$\begin{array}{c} {\dot{x}}_{2}=\alpha_{6}zx_{1}-\alpha_{7}(x_{2}+w_{1}) \end{array}$$ (8c)

$$\begin{array}{c} e=x_{2} \end{array}$$ (8d)

where *z* represents the internal, uncontrollable state, *x*_1_ and *x*_2_ are the external, controllable states, *w*_1_ is the reference velocity to track, and *w*_2_ is the disturbance signal. Furthermore, both *w*_1_ and *w*_2_ are the exogenous signals generated by (2), and μ denotes the control input. This system can be generalized as


$$\begin{array}{c} \dot{z}=\tilde{f}(z,x,w) \end{array}$$ (9a)

$$\begin{array}{c} \dot{x}=\phi (z,x,w)+\gamma (z,x,w)[\nu (\mu )-\chi (w)] \end{array}$$ (9b)

where χ(w) represents a matched uncertainty to be regulated by the servocompensator. The configuration specified in (9) simplifies the output regulation challenge into a state feedback stabilization problem with the disturbance being treated as a matched uncertainty to be compensated.

**Assumption 5**. The system (9b) admits a positive definite, continuously differentiable, and radially unbounded Lyapunov function V(x) such that


$$\begin{array}{c} \frac{\partial V}{\partial x}\gamma (\cdot )=0⟹\frac{\partial V}{\partial x}\phi (\cdot )\;<\;0,\;\forall \;z,x\neq 0 \end{array}$$ (10)

In the framework of optimality, this Lyapunov function is the CLF and, therefore, an optimal value function. Moreover, any Lyapunov function with a time-derivative that is guaranteed to be negative definite meets the criteria for a CLF.

**Remark 5.** Assumption 5 follows directly from the results in [22] where systematic methods are developed for constructing CLFs within the inverse optimal framework.

**Assumption 6.** A locally Lipschitz mapping Ψ(z,x,w), satisfying Ψ(0,0,w)=0, is assumed to exist and together with the CLF defined in Assumption 5, adheres to


$$\begin{array}{c} \beta_{1}(||x||)\leq V(x)\leq \beta_{2}(||x||)\\ \frac{\partial V}{\partial w}S_{0}w+\frac{\partial V}{\partial x}\left[\phi (z,x,w)+\gamma (z,x,w)\;\nu (\Psi (z,x,w))\right]\leq -W(x) \end{array}$$

for all $z,x\in 𝒳\subset {\mathbb{R}}^{n}$, where the domain 𝒳 encompasses the origin, and ∀ w∈𝒲, with *W*(*x*) being a positive definite function, and $\beta_{1}$ and $\beta_{2}$ being class 𝒦 functions.

**Remark 6.** Assumption 6 is typically satisfied by selecting a quadratic CLF, such as $V(x)=\frac{1}{2}x^{2}$, for an asymptotically stable scalar system, and can subsequently be generalized to higher-order systems.

Thus, under Assumption 6, the system (9b) can be reformulated as


$$\begin{array}{cc} \dot{x} & =\phi (z,x,w)+\gamma (z,x,w)\;\nu (\Psi (z,x,w))+\gamma (z,x,w)\;\nu (\mu )\\ & \;-\gamma (z,x,w)[\chi (w)+\nu (\Psi (x,w))] \end{array}$$ (11)

The aim of servocompensation is to develop a control strategy to address the uncertainty [χ(w)+ν(Ψ(x,w))]. This is achieved by designing a suitable conditional servocompensator via the Lyapunov redesign approach. Therefore, a compact set $\Theta =\{\sup_{w\in 𝒲}V(x,w)\leq r\}\subset 𝒳$ for *r* > 0 and a continuous mapping $\delta_{\mu }(z,x)$, uninfluenced by the sector-bounded nonlinearity ν(·), exists which satisfies


$$\begin{array}{c} ||\chi (w)+\nu (\Psi (z,x,w))||\leq \delta_{\mu }(z,x,w)\;\forall \;z,x\in \Theta ,\;\forall \;w\in 𝒲 \end{array}$$ (12)

**Assumption 7.** The term $\frac{\partial V}{\partial x}\gamma (\cdot )$ can be restated as


$$\begin{array}{c} \frac{\partial V}{\partial x}\gamma (\cdot )=b_{\Psi }^{T}(z,x,w)\;\Sigma (z,x,w) \end{array}$$ (13)

where $b_{\Psi }(z,x,w)$ is known a locally Lipschitz function fulfilling $b_{\Psi }(0)=0$ and Σ(z,x,w) is a potentially unknown mapping adhering to


$$\begin{array}{c} 0\leq \Lambda_{1}\leq ||\Sigma (z,x,w)||\leq \Lambda_{2},\;\forall \;z,x\in \Theta ,\;\forall \;w\in 𝒲 \end{array}$$

**Remark 7.** Assumption 7 guarantees that any uncertainty in γ(·) remains bounded in the interval $\left[\Lambda_{1},\Lambda_{2}\right]$. If there is no uncertainty in γ(·), then Σ(·)=I.

**Assumption 8.** For the system $\dot{z}=\tilde{f}(z,\xi ,w,y)$, a candidate Lyapunov candidate V_1_(z) exists, for which


$$\begin{array}{c} \beta_{3}(||z||)\;\leq \;V_{1}(z)\;\leq \;\beta_{4}(||z||)\\ \frac{\partial V_{1}}{\partial z}\tilde{f}(z,\xi ,w,y)+\frac{\partial V_{1}}{\partial w}S_{0}w\;\leq \;-\beta_{5}(||z||)\;\forall \;||z||\geq \beta_{6}(||\xi ||) \end{array}$$

for all $z,\xi ,y\in \Gamma_{0}\subset {\mathbb{R}}^{n}$ and for all w∈𝒲, where $\beta_{3}$ to $\beta_{6}$ are class 𝒦 functions independent of *w*.

**Assumption 9.** Within a vicinity of the zero-error manifold, a Lyapunov function V_2_(z) exists which fulfills the inequality constraints


$$\begin{array}{c} {\tilde{\beta }}_{1}||z|{|}^{2}\;\leq \;V_{2}(z)\;\leq \;{\tilde{\beta }}_{2}||z|{|}^{2}\\ \frac{\partial V_{2}}{\partial z}\tilde{f}(z,\xi ,w,y)+\frac{\partial V_{2}}{\partial w}S_{0}w\;\leq \;-{\tilde{\beta }}_{3}||z|{|}^{2}\\ \left|\left|\frac{\partial V_{2}}{\partial z}\right|\right|\;\leq \;{\tilde{\beta }}_{4}||z|| \end{array}$$

for a set of positive scalars ${\tilde{\beta }}_{1}$ to ${\tilde{\beta }}_{4}$ independent of *w*.

**Remark 8.** Similar to Assumption 6, Assumptions 8 and 9 are commonly satisfied by selecting a quadratic Lyapunov function for asymptotically stable systems.

## 3. State feedback control

We adopt the inverse optimal controller as the stabilizing controller within the state-feedback control framework. The formulation of the state feedback controller incorporating the inverse optimal controller is facilitated through the use of Sontag’s formula together with the foundation laid by Freeman and Kokotović for CLF-based inverse optimality [22]. This facilitates the synthesis of a nonlinear stabilizing feedback control law that also minimizes a meaningful cost functional and embeds optimality within the control framework.

### 3.1. Inverse optimal control

For the nominal representation of the system (9b),


$$\begin{array}{c} \dot{x}=\phi (z,x)+\gamma (z,x)\mu \end{array}$$ (14)

the goal of the optimal control framework is to devise a control law μ=−k(z,x) that minimizes a cost function *J*, also called the optimal value function, expressed as [22]:


$$\begin{array}{c} J=\int_{0}^{\infty }\left[l(z,x)+\mu^{T}R(z,x)\mu \right]\;dt \end{array}$$ (15)

where l(z,x)≥0,R(z,x)>0 ∀ x. Minimizing the optimal value function guarantees the optimal property of the state-feedback stabilizing control law as well as the robustness and stability margins irrespective of the choice of *l*(*z*,*x*) and *R*(*z*,*x*). Hence, if a positive semi-definite function *V*(*x*), the CLF, is taken as the cost function, the inverse optimal prob*l*em can be stabilized by the control input


$$\begin{array}{c} \mu =-\frac{1}{2}R^{-1}(z,x){\left(\frac{\partial V}{\partial x}\gamma (z,x)\right)}^{T} \end{array}$$ (16)

Thus, setting $\mu =-\frac{1}{2}k(z,x)$, the CLF, *V*(*x*), guarantees that


$$\begin{array}{c} l(z,x)=\dot{V}=\left(\frac{\partial V}{\partial x}\phi (z,x)\right)-\frac{1}{2}\left(\frac{\partial V}{\partial x}\gamma (z,x)\right)k(z,x)\leq 0 \end{array}$$ (17)

and solves the HJB equation [22]:


$$\begin{array}{c} l(z,x)+\left(\frac{\partial V}{\partial x}\phi (z,x)\right)-\frac{1}{4}\left(\frac{\partial V}{\partial x}\gamma (z,x)\right)R^{-1}(z,x){\left(\frac{\partial V}{\partial x}\gamma (z,x)\right)}^{T}=0 \end{array}$$

Therefore, if the controller (16) asymptotically stabilizes (14), and if *V*(*x*) satisfies the criteria for (17), then (16) qualifies as an inverse optimal control law. Moreover, this renders the CLF an optimal value function whose a priori choice permits the use of Sontag’s formula [36] for the controller design.

For the nominal external dynamics of the test case (8b)-(8c), a candidate Lyapunov function is given by


$$\begin{array}{c} V(x)=x^{T}Px \end{array}$$ (18)

where *P*, being a positive semi-definite matrix, is a solution to the Riccati inequality


$$\begin{array}{c} A^{T}P+PA-PBB^{T}P<0 \end{array}$$ (19)

Linearizing (8b)-(8c) about the equilibrium points $\left(x_{1}^{*},x_{2}^{*})=(0,-w_{1}\right)$ results in the linear system matrices


$$\begin{array}{c} A=[\begin{array}{cc} -{\tilde{\alpha }}_{4} & 0\\ \frac{{\tilde{\alpha }}_{3}{\tilde{\alpha }}_{6}}{{\tilde{\alpha }}_{1}} & -{\tilde{\alpha }}_{7} \end{array}],\;B=[\begin{array}{c} {\tilde{\alpha }}_{5}\\ 0 \end{array}] \end{array}$$ (20)

where ${\tilde{\alpha }}_{1},\cdots ,{\tilde{\alpha }}_{7}$ represent the nominal values of $\alpha_{1},\cdots ,\alpha_{7}$. Therefore, inequality (19) holds for


$$\begin{array}{c} P=[\begin{array}{cc} p_{1}{\tilde{\alpha }}_{4} & p_{2}{\tilde{\alpha }}_{6}\\ p_{2}{\tilde{\alpha }}_{6} & p_{3}{\tilde{\alpha }}_{7} \end{array}] \end{array}$$

where $p_{1},p_{2},$ and *p*_3_ are selected to ensure the positive semi-definiteness of *P*, yielding


$$\begin{array}{c} V(x)=p_{1}{\tilde{\alpha }}_{4}x_{1}^{2}+2p_{2}{\tilde{\alpha }}_{6}x_{1}x_{2}+p_{3}{\tilde{\alpha }}_{7}x_{2}^{2} \end{array}$$ (21)

which satisfies the conditions of a CLF, thereby ensuring optimality.

**Remark 9.** The matrix A is Hurwitz and the pair (*A,B*) is controllable. This ensures that (19) has a positive semi-definite solution P and validates that the quadratic Lyapunov function (18) qualifies as a CLF.

Therefore, setting


$$\begin{array}{c} \phi (\cdot )=[\begin{array}{c} -{\tilde{\alpha }}_{4}x_{1}\\ {\tilde{\alpha }}_{6}zx_{1}-{\tilde{\alpha }}_{7}(x_{2}+w_{1}) \end{array}],\;\gamma (\cdot )=[\begin{array}{c} {\tilde{\alpha }}_{5}\\ 0 \end{array}] \end{array}$$

yields


$$\begin{array}{cc} \frac{\partial V}{\partial x}\phi (\cdot ) & =\;-2p_{1}({\tilde{\alpha }}_{4}x_{1}{)}^{2}-2p_{3}({\tilde{\alpha }}_{7}x_{2}{)}^{2}-2p_{2}({\tilde{\alpha }}_{4}+{\tilde{\alpha }}_{7}){\tilde{\alpha }}_{6}x_{1}x_{2}\\ & \;+(p_{2}{\tilde{\alpha }}_{6}x_{1}+p_{3}{\tilde{\alpha }}_{7}x_{2})(2{\tilde{\alpha }}_{6}zx_{1}-2{\tilde{\alpha }}_{7}w_{1})=a_{\Psi }(\cdot ) \end{array}$$ (22a)

$$\begin{array}{c} \frac{\partial V}{\partial x}\gamma (\cdot )=\;{\tilde{\alpha }}_{5}(2p_{1}{\tilde{\alpha }}_{4}x_{1}+2p_{2}{\tilde{\alpha }}_{6}x_{2})=b_{\Psi }(\cdot ) \end{array}$$ (22b)

which, when incorporated into the Sontag’s Formula [36] generates the inverse optimal control law as


$$\begin{array}{c} \Psi (\cdot )=-\left[c_{\Psi }+\frac{a_{\Psi }+\sqrt{a_{\Psi }^{2}+[b_{\Psi }^{T}b_{\Psi }{]}^{2}}}{b_{\Psi }^{T}b_{\Psi }}\right]b_{\Psi },\;c_{\Psi }\geq 0,\;b_{\Psi }\neq 0 \end{array}$$ (23)

It is demonstrated in [22] that *V*(*x*) is a CLF satisfying the small control property, rendering the control law (23) Lipschitz continuous at *x* = 0 and optimal stabilizing for the cost function


$$\begin{array}{c} J=\int_{0}^{\infty }\left(\frac{1}{2}\Phi (\cdot )b_{\Psi }^{T}b_{\Psi }+\frac{1}{2\Phi (\cdot )}\Psi^{T}\Psi \right) \end{array}$$

where


$$\begin{array}{c} \Phi (\cdot )=\left\{ \begin{array}{cc} c_{\Psi }+\frac{a_{\Psi }+\sqrt{a_{\Psi }^{2}+{\left[b_{\Psi }^{T}b_{\Psi }\right]}^{2}}}{\left[b_{\Psi }^{T}b_{\Psi }\right]}, & b_{\Psi }(\cdot )\neq 0\\ \;\;c_{\Psi }, & b_{\Psi }(\cdot )=0 \end{array}\right. \end{array}$$

Therefore, for the closed-loop system, this control law results in


$$\begin{array}{cc} \dot{V} & =a_{\Psi }(\cdot )+b_{\Psi }(\cdot )\Psi (\cdot )\\ & =a_{\Psi }-b_{\Psi }\left[b_{\Psi }c_{\Psi }\right]-a_{\Psi }-\sqrt{a_{\Psi }^{2}+[b_{\Psi }^{T}b_{\Psi }{]}^{2}}\\ \dot{V} & =-\left(b_{\Psi }^{2}c_{\Psi }+\sqrt{a_{\Psi }^{2}+{\left[b_{\Psi }^{T}b_{\Psi }\right]}^{2}}\right)\;<\;0,\;x\neq 0 \end{array}$$ (24)

which establishes the system’s global asymptotic stability. Also, together with (24), (18) adheres to the conditions set out in Assumption 5, rendering it an optimal value function and establishing that the inverse optimal stabilizing controller (23) is a globally asymptotically stabilizing control law. Furthermore, (23) guarantees a sector margin $\left(\frac{1}{2},\infty \right)$ for the nominal system of (8b)-(8d). Additionally, based on the nature of the chosen CLF, it also guarantees disk margin $𝒟\left(\frac{1}{2}\right)$ [22,37].

### 3.2. Conditional servocompensator

In order to proceed with output regulation, a conditional servocompensator is integrated into the control architecture via a saturated high-gain feedback controller, leveraging the Lyapunov redesign framework to effectively resolve the servomechanism problem for time-dependent references and disturbances. [14].

The design of the servocompensator is initiated by applying (7) to (8) which leads to


$$\begin{array}{cc} \frac{\partial \Pi (w)}{\partial w}S_{0}w & =[\begin{array}{c} -{\tilde{\alpha }}_{1}\Pi_{1}-{\tilde{\alpha }}_{2}\Pi_{2}(\Pi_{3}+w_{1})+{\tilde{\alpha }}_{3}\\ -{\tilde{\alpha }}_{4}\Pi_{2}+{\tilde{\alpha }}_{5}(w_{2}+\chi (w))\\ {\tilde{\alpha }}_{6}\Pi_{1}\Pi_{2}-{\tilde{\alpha }}_{7}(\Pi_{3}+w_{1}) \end{array}]\\ h(\Pi ,w) & =\Pi_{3}-w_{1}=0 \end{array}$$ (25)

Solving (25) determines the steady-state control as


$$\begin{array}{c} \chi (w)=\omega w_{3}-w_{2}+\frac{{\tilde{\alpha }}_{1}{\tilde{\alpha }}_{4}}{{\tilde{\alpha }}_{3}{\tilde{\alpha }}_{5}{\overset{˘}{\alpha }}_{6}} \end{array}$$ (26)

which defines the matched uncertainty to be regulated by the servocompensator. Therefore, setting $q=3,\vartheta_{0}=\vartheta_{1}=\omega^{2}$, and $\vartheta_{2}=0$ in (5) satisfies the characteristic polynomial


$$\begin{array}{c} {ℒ}_{s}^{3}\chi =-\omega^{2}{ℒ}_{s}\chi \end{array}$$ (27)

signaling the existence of a quadratic nonlinearity to be addressed by the servocompensator. Accordingly, expressing the matrices *S* and *J* as


$$\begin{array}{c} S=[\begin{array}{ccc} 0 & 1 & 0\\ 0 & 0 & 1\\ 0 & -\omega^{2} & 0 \end{array}],\;J=[\begin{array}{c} 0\\ 0\\ 1 \end{array}] \end{array}$$

enables the formulation of a third-order conditional servocompensator specified as


$$\begin{array}{c} \dot{\varsigma }=(S-JK)\varsigma +\rho \;sat\left(\frac{s}{\rho }\right) \end{array}$$ (28)

where ς denotes the output produced by the conditional servocompensator, 0≤ρ≤1 denotes the width of the boundary layer, $s=b_{\Psi }(\cdot )+K\varsigma$ is the sliding surface, and the saturation function is defined as


$$\begin{array}{c} sat\left(\frac{s}{\rho }\right)=\left\{ \begin{array}{cc} \frac{s}{\left||s|\right|}, & ||s||\geq \rho \\ \frac{s}{\rho }, & ||s||\leq \rho \end{array}\right. \end{array}$$

Additionally, the pair (*S*, *J*) is controllable and the gain matrix *K* is selected to ensure that S−JK is Hurwitz.

### 3.3. State feedback control design

The resulting full-state feedback control law combines the inverse optimal stabilizing controller with the conditional servocompensator, embedded within a saturated high-gain feedback structure. Accordingly, we can formulate the state feedback control law as


$$\begin{array}{c} \mu =-a_{\mu }(z,x,w)\;sat\left(\frac{s}{\rho }\right) \end{array}$$ (29)

where $a_{\mu }(z,x,w)$ is a continuous function satisfying


$$\begin{array}{c} a_{\mu }(z,x,w)\;\geq \;\delta_{\mu }(z,x,w)+a_{0},\;a_{0}>0 \end{array}$$ (30)

Following Assumption 6, setting


$$\begin{array}{c} a_{\mu }(\cdot )=-(||\Psi (\cdot )+\chi (w)||+a_{0}) \end{array}$$ (31)

the state feedback controller can be expressed as


$$\begin{array}{c} \mu =-(||\Psi (\cdot )+\chi (w)||+a_{0})\;sat\left(\frac{s}{\rho }\right) \end{array}$$ (32)

This control law inherits all the optimality and robustness properties of the inverse optimal stabilizing feedback controller Ψ(·) as well as providing robust output regulation under the influence of sector-bounded nonlinearity ν(·).

A detailed analysis of the closed-loop stability and performance of the controller is presented in [15].

## 4. Output feedback control

The state feedback controller (32) requires access to the complete system dynamics, a limitation imposed by the structure of the stabilizing controller. This, however, is often infeasible in practical implementations where only partial state measurements are available. To overcome this limitation, we now extend the design to an output feedback framework by employing a full-order high-gain observer (HGO) to reconstruct the unmeasured states.

The full-order observer design integrates two observers: a partial-state observer to approximate the internal state(s) of the system [38], and an HGO to approximate the external state(s) of the system. We work under the assumption that only the motor’s angular velocity is available for feedback. Therefore, we first design a partial-state observer to reconstruct the internal states of the system.

For the system (8), Assumptions 8 and 9 are satisfied using $V_{1}=V_{2}=\frac{1}{2}z^{2}$ and demonstrate the exponential convergence of the state to the zero-error manifold, enabling the characterization of the partial-state observer as


$$\begin{array}{c} \dot{\hat{z}}=-{\tilde{\alpha }}_{1}\hat{z}-{\tilde{\alpha }}_{2}yx_{1}+{\tilde{\alpha }}_{3} \end{array}$$ (33)

Subsequently, for the design of the HGO, the external dynamics, $[x_{1},x_{2}{]}^{T}$ must first be transformed into the normal form by employing the transformation matrix


$$\begin{array}{c} T(\cdot )=[\begin{array}{c} \xi_{1}\\ \xi_{2} \end{array}]=[\begin{array}{c} x_{2}\\ {\overset{˘}{\alpha }}_{6}zx_{1}-{\overset{˘}{\alpha }}_{7}x_{2} \end{array}] \end{array}$$ (34)

This facilitates the design of the HGO as


$$\begin{array}{cc} {\dot{\hat{\xi }}}_{1} & ={\hat{\xi }}_{2}+\frac{g_{1}}{\varepsilon }(y_{\xi }-{\hat{\xi }}_{1})\\ {\dot{\hat{\xi }}}_{1} & =\frac{g_{2}}{\varepsilon^{2}}(y_{\xi }-{\hat{\xi }}_{1})\\ y_{\xi } & =\xi_{1} \end{array}$$ (35)

where *g*_1_ and *g*_2_ are scalar parameters selected to ensure that the roots of the characteristic polynomial


$$\begin{array}{c} \lambda^{2}+g_{1}\lambda +g_{2}=0 \end{array}$$

are located strictly in the open left-half plane of the complex plane and ε∈(0,1) is the observer gain parameter.

The full-order observer is then formulated as


$$\begin{array}{cc} \dot{\hat{z}} & =-{\tilde{\alpha }}_{1}\hat{z}-{\tilde{\alpha }}_{2}y{\hat{x}}_{1}+{\tilde{\alpha }}_{3}\\ {\dot{\hat{\xi }}}_{1} & ={\hat{\xi }}_{2}+\frac{g_{1}}{\varepsilon }(y_{\xi }-{\hat{\xi }}_{1})\\ {\dot{\hat{\xi }}}_{1} & =\frac{g_{2}}{\varepsilon^{2}}(y_{\xi }-{\hat{\xi }}_{1}) \end{array}$$ (36)

and the resulting reconstructed state vector $\left[\hat{z},{\hat{x}}_{1},{\hat{x}}_{2}\right]$ is used with (32) to formulate the output feedback controller


$$\begin{array}{cc} \dot{\varsigma } & =(S-JK)\varsigma +\rho \;sat\left(\frac{\hat{s}}{\rho }\right)\\ \mu & =-(||\Psi (\hat{z},\hat{x},w)+\chi (w)||+a_{0})\;sat\left(\frac{\hat{s}}{\rho }\right)\\ \hat{s} & ={\hat{b}}_{\Psi }(\cdot )+K\varsigma \end{array}$$ (37)

where ${\hat{b}}_{\Psi }(\cdot )$ denotes the estimated approximation of $b_{\Psi }(\cdot )$.

## 5. Analysis

The stability analysis of the closed-loop system and its ability to recover performance are henceforth demonstrated. The analysis builds on the foundational framework presented in [15] and validates that, with the incorporation of observer dynamics, the overall system behavior remains robust and consistent with theoretical expectations.

To analyze the performance recovery of the HGO, consider the scaled estimation errors for $z\in {\mathbb{R}}^{1}$ and $\xi \in {\mathbb{R}}^{2}$ as $\eta_{z}=z-\hat{z}$, $\eta_{1}=(\xi_{1}-{\hat{\xi }}_{1})/\varepsilon$, and $\eta_{2}=\xi_{2}-{\hat{\xi }}_{2}$, respectively, and differentiate them with respect to time to obtain


$$\begin{array}{cc} \dot{\eta_{z}} & =A_{z}\eta_{z}+\Delta_{z}(\cdot )\\ \varepsilon \dot{\eta } & =A_{\eta }\eta +\varepsilon B_{\eta }\Delta (\cdot ) \end{array}$$ (38)

where $\Delta_{z}(\cdot )=\tilde{f}(\cdot )-{\tilde{f}}_{0}(\cdot )$ and $\Delta (\cdot )=\gamma (\cdot )-\gamma_{0}(\cdot )$. Combining (8), (37) and (38) via (34) enables the formulation of the standard singularly perturbed system where $\left(\xi ,\varsigma ,\eta_{z}\right)$ denotes the slow variable obtained by setting ε=0, and η denotes the fast variable. It has been demonstrated in [38] that for sufficiently small ρ, every trajectory in the slow model, ${\left[\dot{\xi },\;\dot{\varsigma },\;\dot{\eta_{z}}\right]}^{T}$, exponentially approaches the invariant zero-error manifold.

Furthermore, consider the fast model, $\dot{\eta }$, where, under the assumptions outlined in [28], the system matrices are given by


$$\begin{array}{c} A_{\eta }=[\begin{array}{cc} -\frac{g_{1}}{\varepsilon } & 1\\ -\frac{g_{2}}{\varepsilon^{2}} & 0 \end{array}],\;B_{\eta }=[\begin{array}{c} 0\\ 1 \end{array}],\;\Delta (\cdot )\;\leq \;L||\eta ||+M \end{array}$$

where *L* and *M* are positive constants. Setting ε=0 effectively eliminates the impact of Δ(·). Next, consider the Lyapunov candidate function


$$\begin{array}{c} V_{\eta }=\varepsilon \eta^{T}P_{\eta }\eta \end{array}$$ (39)

where $P_{\eta }>0$ is a symmetric matrix computed using $P_{\eta }A_{\eta }+A_{\eta }^{T}P_{\eta }=-I$, where $I\in {\mathbb{R}}^{2}$ is the identity matrix. Differentiating (39) with respect to time yields


$$\begin{array}{cc} {\dot{V}}_{\eta } & ={\dot{\eta }}^{T}P_{\eta }\eta +\eta^{T}P_{\eta }\dot{\eta }\\ & =-\eta^{T}\eta +2\varepsilon B_{\eta }P_{\eta }\Delta (\cdot )\eta \\ {\dot{V}}_{\eta } & \leq -||\eta ||\left[(1-2\varepsilon L||B_{\eta }P_{\eta }||)||\eta ||-2\varepsilon M||B_{\eta }P_{\eta }||\right] \end{array}$$ (40)

which establishes that


$$\begin{array}{c} \dot{V_{\eta }}<0\;\forall \;||\eta ||>\frac{2\varepsilon M\left||B_{\eta }P_{\eta }|\right|}{1-2\varepsilon L||B_{\eta }P_{\eta }||} \end{array}$$

indicating that the steady-state error decreases as ε decreases, resulting in higher estimation accuracy for small values of ε.

The aforementioned analysis can be generalized to $\eta_{z}\in {\mathbb{R}}^{n-l}$ and $\eta \in {\mathbb{R}}^{l}$.

Consequently, the closed-loop system can be represented as


$$\begin{array}{cc} \dot{w} & =S_{0}w\\ \dot{\varsigma } & =(S-JK)\varsigma +\rho \;sat\left(\frac{\hat{s}}{\rho }\right)\\ \dot{\hat{z}} & =A_{z}\;z+B_{z}\;{\tilde{f}}_{0}(\hat{z},\hat{\xi },w,y)\\ \dot{\hat{\xi }} & =A_{\xi }\;\hat{\xi }+B_{\xi }\;[\phi_{0}(\hat{z},\hat{\xi },w)+\gamma_{0}(\mu +w)]+\Lambda_{\xi }(y_{\xi }-C\hat{\xi }) \end{array}$$ (41)

Based on the preceding formulation and analysis, the main result is formally presented in the subsequent theorem.

**Theorem 1.** *Consider the closed-loop system (41) and suppose* w(0)∈𝒲*. Then, using Assumptions 1–9, there exists* $\rho^{*}>0$ *which guarantees that for every* $\rho \in (0,\rho^{*}]$*, a corresponding* $\varepsilon^{*}=\varepsilon^{*}(\rho )>0$ *exists for which all system trajectories remain bounded and the tracking error satisfies* $\lim_{t\to \infty }||y(t)-w(t)||=0\;\forall \;\varepsilon \in (0,\varepsilon^{*}(\rho )]$*. Furthermore, for every* $\delta_{0}>0$*, a corresponding* $\rho_{1}^{*}>0$ *exists and for each* $\rho \in (0,\rho_{1}^{*}]$*, there exists a* $\varepsilon_{1}^{*}=\varepsilon_{1}^{*}(\rho )>0$ *which ensures* $\forall \;\varepsilon \in (0,\varepsilon_{1}^{*}(\rho )]$*,*


$$\begin{array}{c} \left|\left|[\begin{array}{c} \hat{z}(t)\\ \hat{x}(t) \end{array}]-[\begin{array}{c} z(t)\\ x(t) \end{array}]\right|\right|\;\leq \;\delta_{0}\;\forall \;t\;\geq \;0 \end{array}$$

**Remark 10.** The systematic integration of the inverse optimal controller with a conditional servocompensator and HGO yields distinct properties. Specifically, the HGO reconstructs the unmeasured state dynamics from system, while the inverse optimal control law embeds native properties of optimality and robust margins during stabilization. Furthermore, as mathematically formalized in Theorem 1, the overall stability of the interconnected closed-loop system is verified through a singular perturbation approach. The analysis guarantees that the fast state-estimation error dynamics are compatible with the convergence of the slow tracking error coordinates, ensuring overall bounded trajectories during transients and asymptotic regulation at steady state.

## 6. Results

We now implement the proposed controller on MATLAB/Simulink using Simscape plant models and present the results. We apply the proposed control law (37) on the system (1) under the effect of disturbances, using the CLF and derivatives defined in Eq (18) and Eq (22), respectively, with *P* set as


$$\begin{array}{c} P=[\begin{array}{cc} 50{\overset{˘}{\alpha }}_{4} & 0.1{\overset{˘}{\alpha }}_{6}\\ 0.1{\overset{˘}{\alpha }}_{6} & 0.005{\overset{˘}{\alpha }}_{7} \end{array}] \end{array}$$ (42)

Furthermore, the matrix *K* is selected such that the poles of S−JK are placed at −0.5,−1, and −1.5. To account for physical hardware limitations, the control effort is constrained via a hard actuator saturation at 240 V, representing realistic energy constraints and eliminating the infinite control effort commonly observed in idealized theoretical simulations. The nominal parameter values of the system, representing a standard separately-excited DC motor model usually used in control literature, are listed in Table 1.

**Table 1:** Nominal System Parameters.

Parameter	Value	Parameter	Value	Parameter	Value
$R_{a}$	0.6 Ω	$L_{a}$	0.012 H	$v_{a}$	240 V
$R_{f}$	240 Ω	$L_{f}$	120 H	$c_{1},\;c_{2}$	1.8 Nm/A
*c*_3_	0.02 Nms	*J*	1 kgm$s^{2}$	$\omega_{ref_{1}}$	90 rad/s
ω	2 rad/s	*d*_0_	5	$\omega_{ref_{2}}$	120 rad/s
$\alpha_{0}$	100	ρ	0.1	ε	$1\times 10^{-3}$
*g*_1_	1	*g*_2_	10		

**Fig 2:**
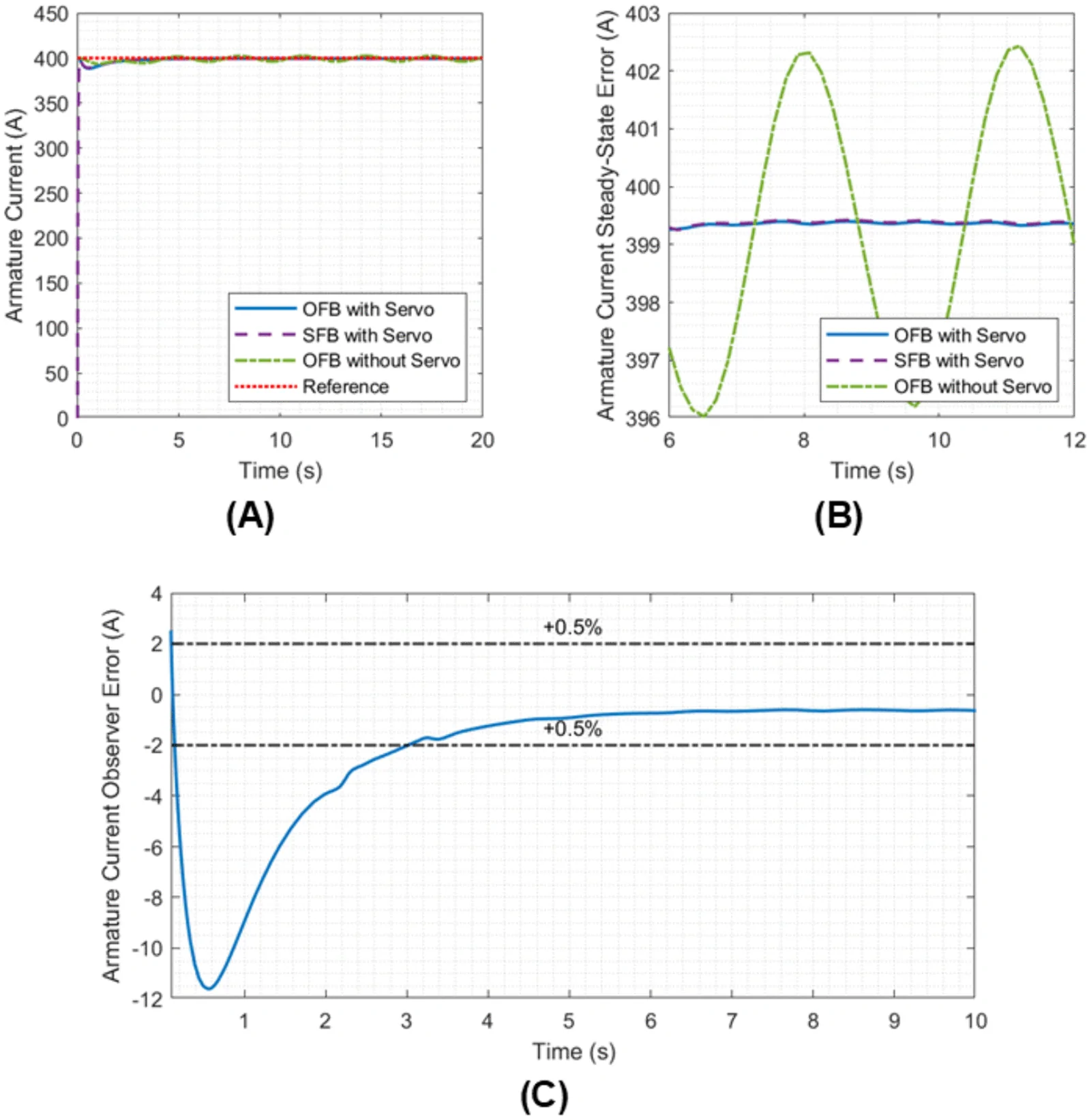
Armature Current. (A) Complete state trajectory. (B) State-state trajectory. (C) Observer error.

**Fig 3:**
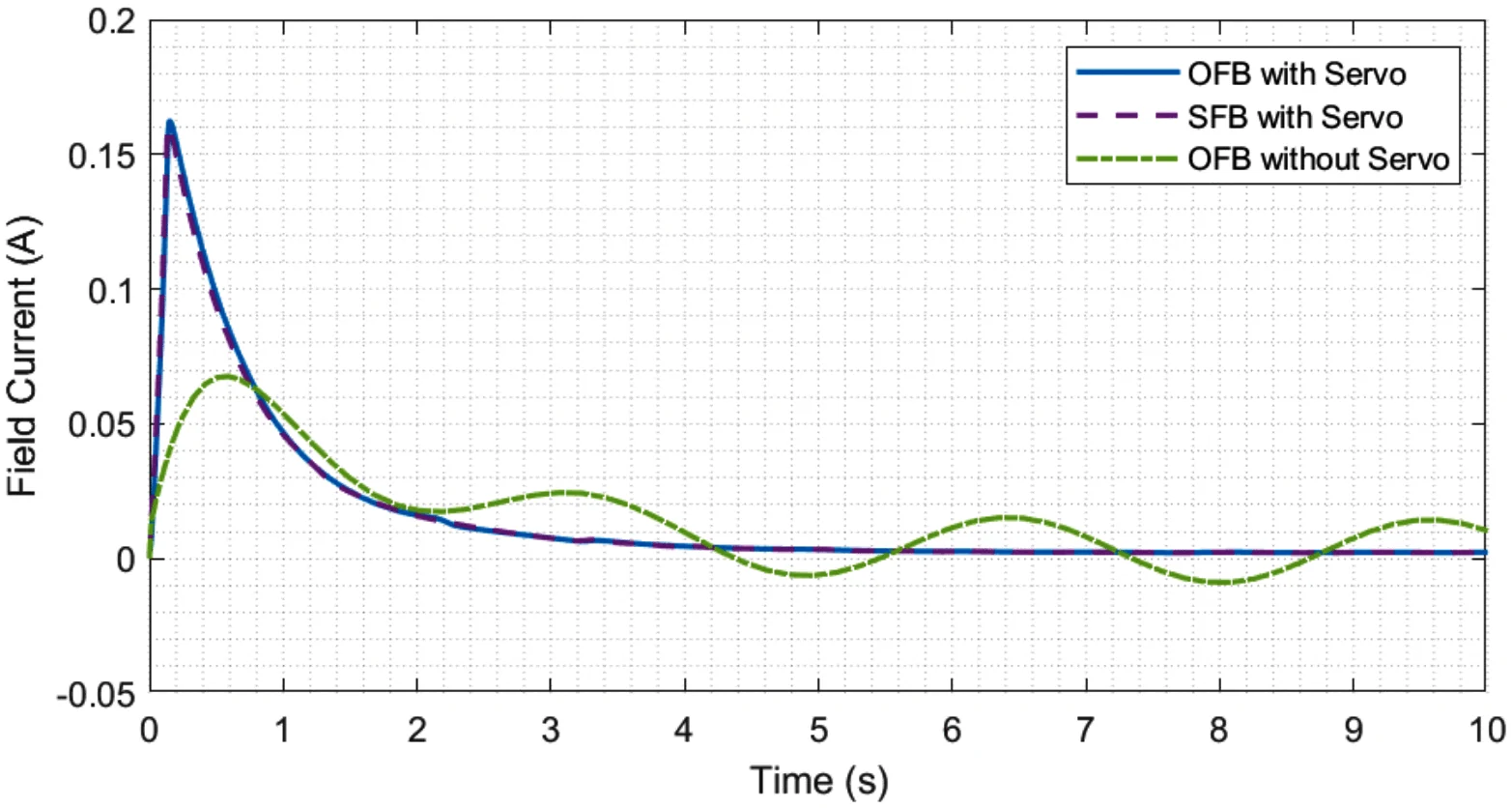
Field Current.

**Fig 4:**
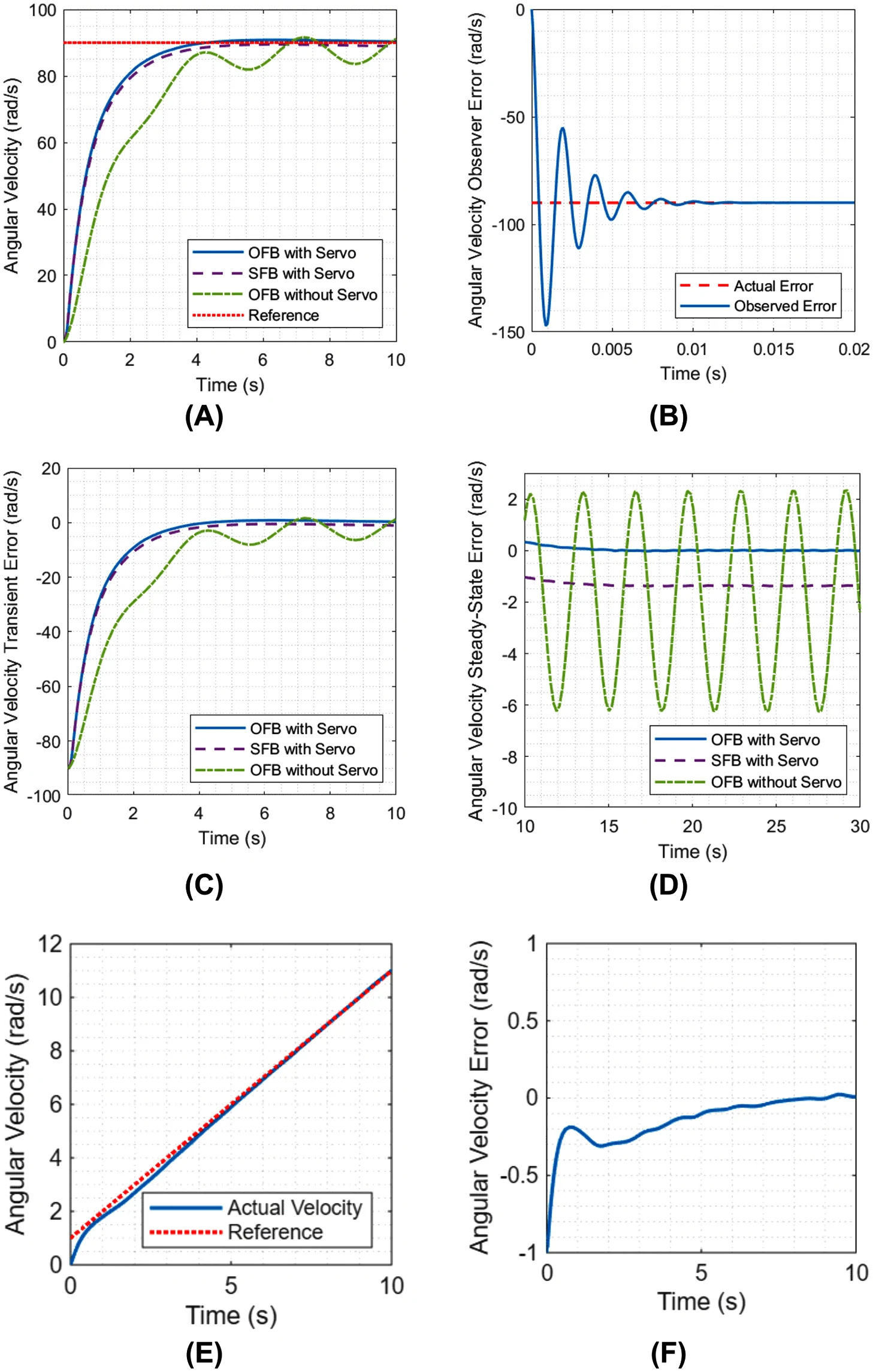
Angular Velocity. (A) Complete state trajectory. (B) Error trajectory. (C) Angular velocity transient state error. (D) Angular velocity steady-state error. (E) Complete state response for ramp input. (F) Response error for ramp input.

Fig 2–4 illustrate the simulation results of the various states under three control schemes: output feedback without servocompensator (OFB without Servo), state feedback with servocompensator (SFB with Servo), and output feedback with servocompensator (OFB with Servo).

Fig 2a depicts the behavior of the internal dynamics, that is, the armature current under the three control schemes. The armature current is not directly influenced by the control input, but evolves according to the closed loop dynamics. It can be observed that all three controllers result in a similar trajectory and the armature current attains its expected value swiftly and accurately, with minute steady-state errors as indicated by Fig 2b. Additionally, Fig 2b also indicates that the servocompensator helps to mitigate the effects of the disturbance. Moreover, Fig 2c illustrates that observed armature current swiftly converges to the actual armature current and the error between the actual and observed currents remains within an acceptable bound of 0.5% for all time.

Likewise, Fig 3 presents similar results for the field current under the application of the three controllers.

Fig 4a-4d compare the impact of the three controllers on the angular velocity of the motor, that is, the output of the system. Fig 4a shows the angular velocity of the motor under no load condition. It is evident that, under the effect of all three controllers, the actual motor velocity swiftly and accurately converges to the desired reference velocity. Moreover, the controllers incorporating servocompensators provide an additional advantage of asymptotically tracking the reference at steady-state, effectively canceling out the disturbance from the exosystem. This is further substantiated by Fig 4c and Fig 4d which illustrate the trajectories of the transient and steady-state errors of the angular velocity, respectively. Fig 4d further highlights that the output feedback control scheme yields better steady-state error performance than its state feedback counterpart.

Fig 4b compares the actual angular velocity error trajectory with the estimated trajectory. It can be seen from the figure that the estimated error rapidly converges to the actual error trajectory, demonstrating the accuracy and effectiveness of the observer in capturing the system dynamics.

Furthermore, the performance of the proposed controller was evaluated under the influence of a ramp reference input to analyze its tracking capabilities for time-varying references. As illustrated in Fig 4e and 4f, the controller successfully tracks the ramp signal while simultaneously rejecting sinusoidal disturbances. The results demonstrate that the controller maintain zero steady-state error, highlighting its efficacy in tracking complex trajectories.

**Fig 5:**
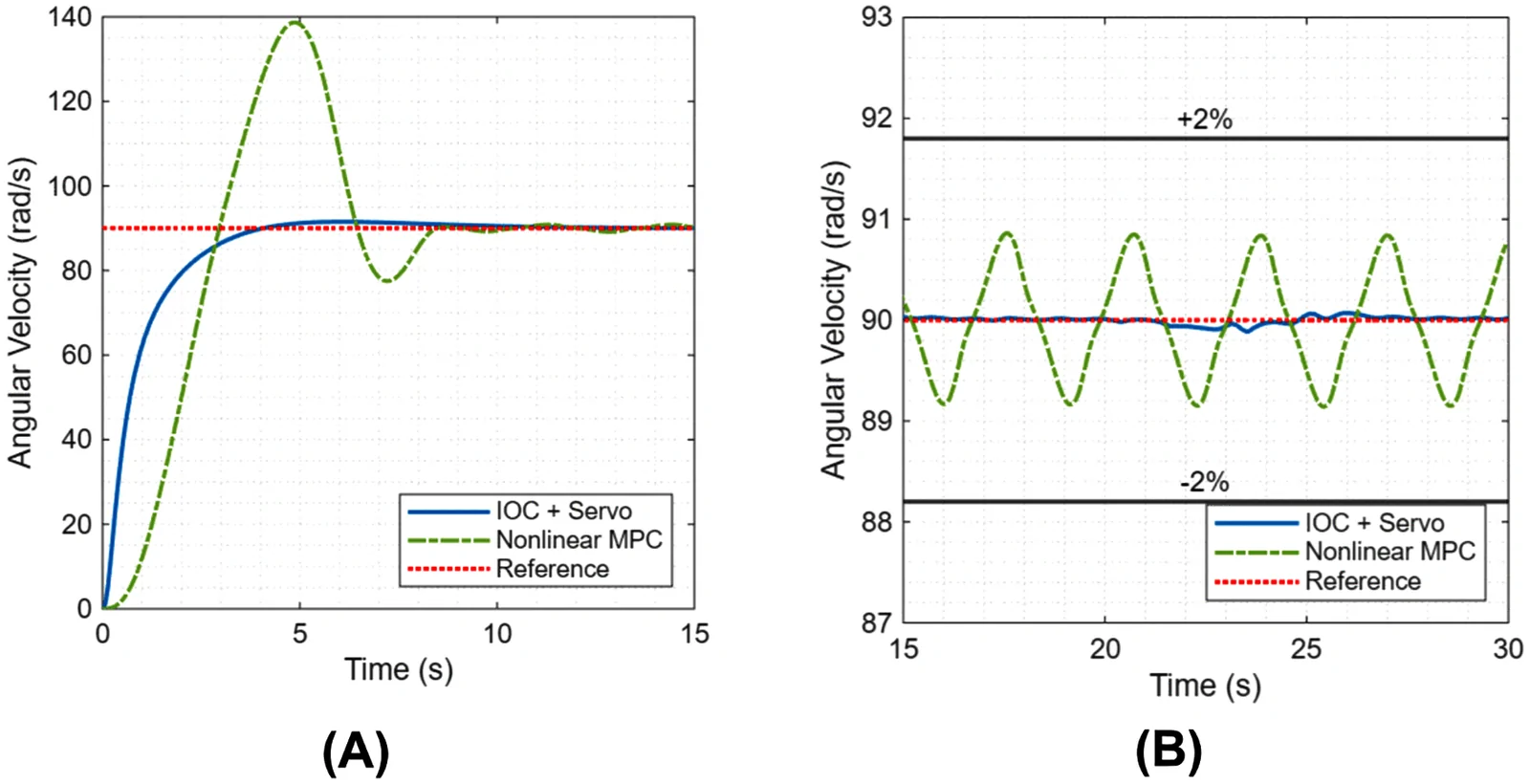
Performance comparison between the proposed controller and a nonlinear MPC. (A) Transient state. (B) Steady-state.

Furthermore, the performance of the proposed controller was evaluated against a state-of-the-art nonlinear MPC with the comparative the results presented in Fig 5. NMPC was selected because the field-controlled DC motor exhibits highly coupled, nonlinear dynamics that degrade the performance of standard linearized predictive models under wide operating conditions. It can be observed from Fig 5a and 5b that while the NMPC successfully stabilizes the system, it exhibits significant transient overshoots and persistent steady-state oscillations under the exogenous disturbance. This performance degradation occurs because the NMPC optimizes the control law over a finite-time horizon, and unless its weights are aggressively tuned, it results in a small, persistent tracking ripple at steady-state. In comparison, the proposed controller embedding the infinite-horizon conditional servocompensator provides superior stabilization and regulation performance by eliminating the persistent disturbance forces.

**Fig 6:**
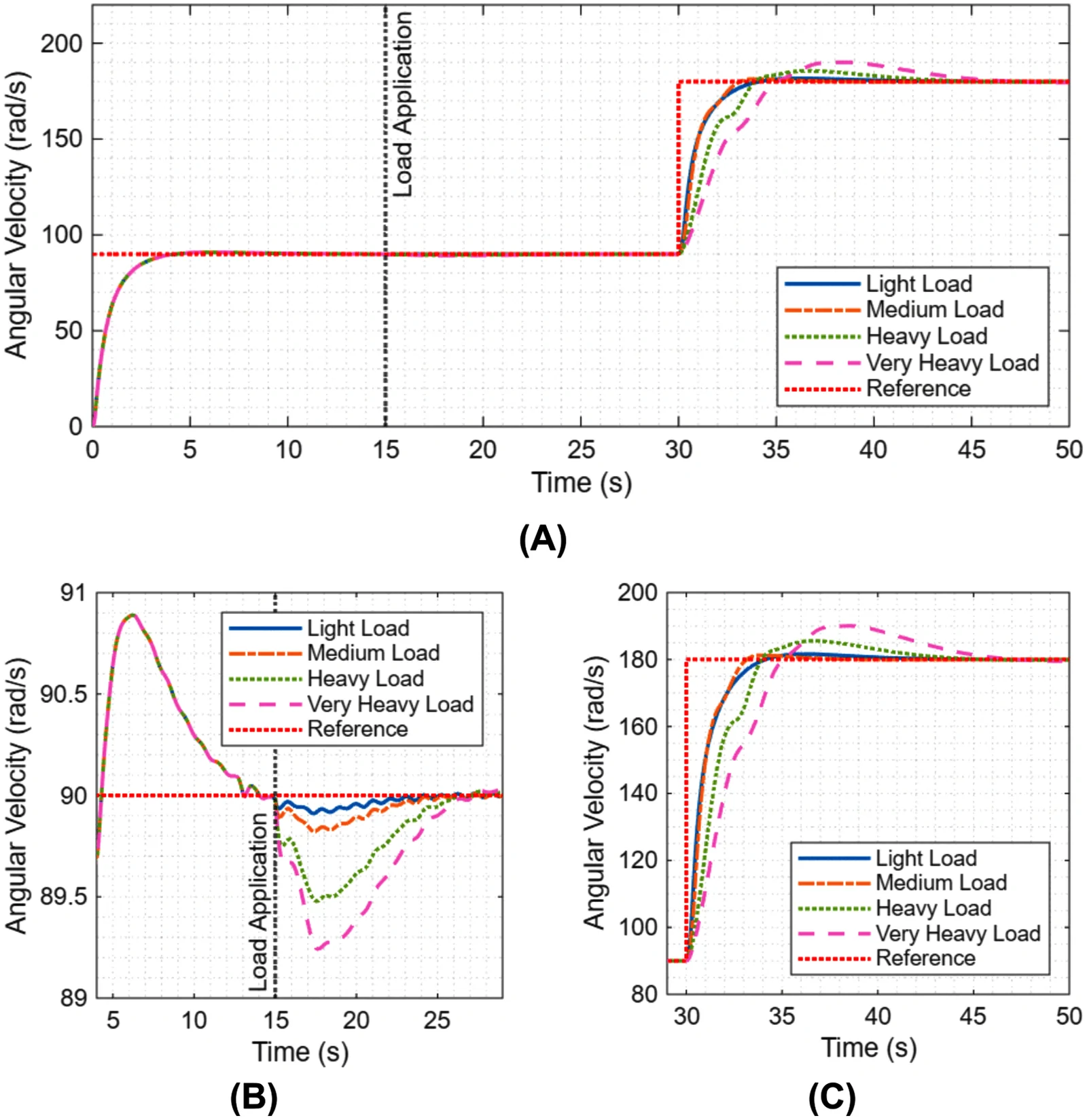
Controller performance under inertial loads. (A) Angular velocity complete trajectory. (B) Trajectory under load variations. (C) Trajectory under reference variations.

Additionally, to evaluate the controller’s robustness against unmodeled mechanical dynamics and load disturbances, we have subjected the controller to variations in inertial load. Inertia values of 0.5, 1, 3, and 5 kgm^2^ were used to represent light, moderate, heavy, and very heavy mechanical couplings, respectively, representing a wide range of operating conditions usually observed in industrial DC drives, mimicking the impact of structural uncertainties and variable payload dynamics. Fig 6 presents the effect of inertial load application and reference changes on the motor speed. In particular, Fig 6b zooms in to illustrate the effect of connecting an inertial load to the motor, while Fig 6c zooms in to depict the effect of changing the reference speed while the inertial load is connected to the motor. Together, these results indicate that the controller effectively compensates for both inputs and maintains asymptotic regulation despite sudden shifts in the plant’s physical parameters.

**Fig 7:**
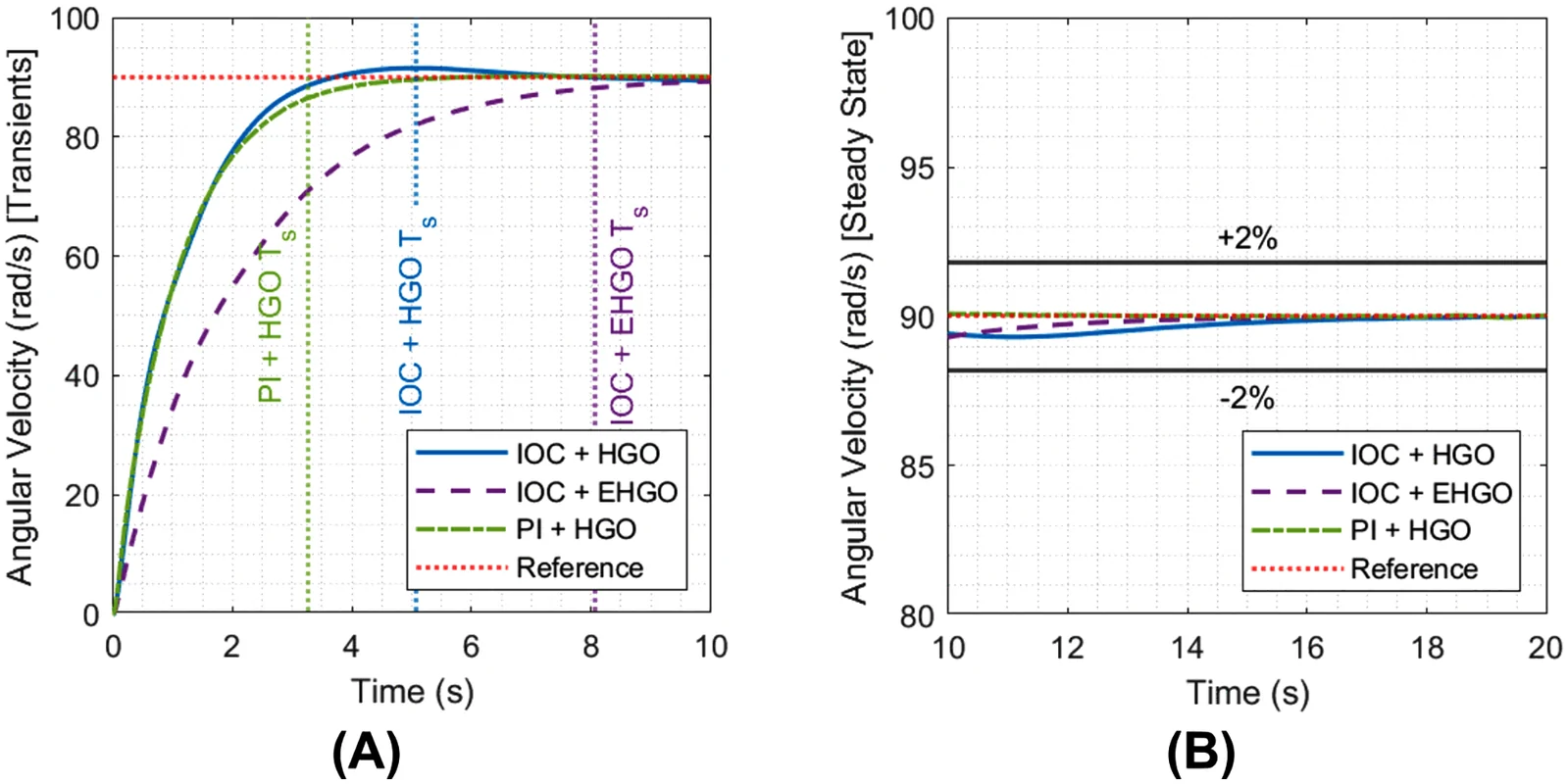
Mean Angular Velocity trajectories for *n* = 250 randomized trials. (A) Transient state. (B) Steady-state.

Finally, we conduct a comprehensive Monte-Carlo robustness analysis with *n* = 250 randomized trials to evaluate the effects of parametric uncertainties and sensor tolerances. The trials involve simultaneous randomization of all plant parameters within a ±20% variance, evaluating the controller’s performance across statistically wide range of non-ideal operating conditions. The results are compared to two baseline configurations: (i) a previously presented control scheme utilizing the inverse optimal controller with the EHGO [39], and (ii) a PI controller integrated with a HGO. The selection of PI controller as the baseline stabilizing controller is due to its widespread use in practical applications, attributed to its simplicity and ease of tuning. The controller performance is compared using five metrics: (i) settling time $\left(T_{s}\right)$, (ii) peak overshoot, (iii) root mean square (RMS) error, (iv) integral absolute error (IAE) at steady state, and (v) computation time. The results are summarized in Table 2 with supporting illustrations provided in Fig 7, and provide a statistical guarantee of robustness against variations often encountered in real-world industrial environments.

**Table 2:** Controller performance under ±20% parametric variations (Mean ± Std Dev).

Control Scheme	IOC + HGO	IOC + EHGO	PI + HGO
$T_{s}$ (s)	5.08 ± 3.56	8.4 ± 1.91	3.263 ± 0.08
Peak OS (%)	2.94 ± 4.32	1.16 ± 9.17	0.3 ± 0.15
RMS Error (rad/s)	0.54 ± 0.14	1.02 ± 3.52	0.27 ± 0.03
IAE (SS) (rad)	4.84 ± 1.55	8.00 ± 31.96	2.12 ± 0.61
Computation Time	0.92 s	1.65 min	0.86 s

The proposed control scheme consistently outperformed the EHGO-based control scheme across all key performance metrics. This demonstrates its feasibility as a practical and computationally efficient controller particularly for systems such as the DC motor. This performance advantage extends to other systems with similar nonlinear dynamics such as quadrotors, robotic manipulators, and electrohydraulic actuators, and more.

When compared to the PI + HGO controller, the proposed control scheme exhibits comparable performance. A minor performance trade-off is observed, which is outweighed by the systematic robustness guarantees and optimality provided by the IOC framework, which allows generalization to a broader class of nonlinear systems. In contrast, the PI-based approach lacks formal guarantees of stability, optimality, or robustness under static and dynamic uncertainties, and requires empirical tuning when applied to other nonlinear systems, limiting its applicability and reliability in more complex and uncertain conditions.

## 7. Conclusion

This study presents an effective and streamlined strategy for solving the output regulation problem by integrating a full-order HGO with an inverse optimal controller and a conditional servocompensator, formulated within the Lyapunov redesign framework. The proposed control scheme, validated via simulations on a DC motor, demonstrated asymptotic stability, improved transient response, and precise regulation under time-varying disturbance conditions.

Notably, the controller relies on a single measured output, the motor speed, to estimate both the internal and external motor dynamics. This addresses the key limitation of requiring full-state availability in inverse optimal control framework. Furthermore, the proposed design also leverages the inherent optimality and robustness properties of the controller and was shown to effectively recover performance following load perturbations and shifts in operating conditions.

The efficacy of the control scheme was further demonstrated through robustness tests with ±20% parametric variations. Notably, the performance of the proposed controller exceeded that of the EHGO-based controller presented in [39], while remaining comparable to a PI-based baseline. However, the minor trade-off offered by PI-based controller is outweighed by the robustness and optimality guarantees inherent to the IOC-based framework. These results support the practical feasibility of the proposed controller for high-performance control tasks, particularly for nonlinear minimum-phase systems.

The partial-state observer employed in the controller design is an open-loop observer for the internal system states and assumes that the estimation error for all unobservable states remains sufficiently small. This assumption simplifies the control architecture, but restricts the applicability of the observer to systems exhibiting minimum phase behavior [38,40]. While not restrictive for the system under study, this approach may not generalize to more complex systems, particularly non-minimum phase systems, where unobservable dynamics may introduce large estimation errors or even destabilize the overall dynamics. Future work may address this limitation and investigate the controller’s viability and performance in more challenging scenarios such as for more complex systems or systems with severe measurement noise.
